# Evolution of an ovarian endometriotic cyst into clear cell carcinoma with squamous differentiation across two pregnancies: A case report

**DOI:** 10.1097/MD.0000000000045418

**Published:** 2025-10-17

**Authors:** Yong Wang, Quan Chen, Yinxia Su, Fei Liu

**Affiliations:** a Department of Pathology, Hubei Provincial Hospital of Traditional Chinese Medicine, Wuhan, China; b Hubei Shizhen Laboratory, Wuhan, China; c Hospital Department, Hubei University of Chinese Medicine, Wuhan, China.

**Keywords:** endometrioid carcinoma, endometriotic cyst, ovarian clear cell carcinoma (OCCC), ovarian tumor, pregnancy

## Abstract

**Rationale:**

Ovarian clear cell carcinoma (OCCC) during pregnancy is exceedingly rare, particularly when accompanied by squamous differentiation, with only a limited number of cases reported in the English literature to date.

**Patient concerns:**

A 33-year-old female, during the first pregnancy, identified endometriotic cysts. However, during the second pregnancy, the patient was subsequently diagnosed with OCCC.

**Diagnoses:**

In this case, histological examination reveals a tumor demonstrating tubular cystic, papillary, and solid growth patterns. Glandular areas with extensive squamous differentiation are observed, along with papillary regions containing hyalinized fibrovascular cores. Tumor cells exhibit cuboidal morphology with clear cytoplasm and hobnail appearance, showing significant atypia. The periphery demonstrates endometrial endometriosis progressing to atypical endometriosis and subsequent malignant transformation. Immunohistochemical analysis shows positive expression of P504S and NapsinA in tumor cells, thereby excluding ovarian endometrioid carcinoma. The findings are consistent with a definitive diagnosis of OCCC.

**Interventions:**

Following the detection of the ovarian mass, left adnexectomy was performed. After definitive diagnosis of OCCC, pregnancy termination was pursued, followed by total hysterectomy, right adnexectomy, and pelvic lymph node dissection.

**Outcomes:**

Postoperative follow-up at 6 months revealed no evidence of recurrence.

**Lessons:**

This case highlights an ovarian cyst that measured 8 cm during the second pregnancy but was only 2 cm in diameter during the first pregnancy, ultimately diagnosed as clear cell carcinoma. Although ovarian malignancies during pregnancy are rare, benign-appearing cysts should not be overlooked. Increased vigilance is warranted to ensure timely diagnosis and appropriate management of such rare but clinically significant presentations.

## 1. Introduction

Ovarian clear cell carcinoma (OCCC) is an epithelial ovarian cancer subtype, accounting for 5% to 10% of all epithelial ovarian tumors.^[1]^ Malignancies arising during pregnancy are exceptionally rare, with an incidence of 0.075% to 0.110%.^[2]^ Although pregnancy-associated ovarian cancers are uncommon, they generally exhibit a more favorable prognosis compared to ovarian malignancies in non-pregnant patients.^[3]^ Epidemiological studies suggest an inverse correlation between multiparity and ovarian cancer risk, with reduced incidence and delayed onset observed in multiparous women.^[3]^ In this case, OCCC with squamous differentiation, morphologically resembling endometrioid carcinoma, is diagnosed during the patient’s second pregnancy. Pathologic differentiation between these entities is critical. Notably, an ovarian endometriotic cyst was initially suspected via ultrasound during the first pregnancy, whereas OCCC was confirmed histologically in the second pregnancy. Microscopic examination reveals the progression from endometriosis to atypical endometriosis and, ultimately, to OCCC, posing diagnostic challenges. This work systematically reviews the clinical, histologic, and immunophenotypic features, along with differential diagnoses and prognostic implications, supported by a literature review. These findings enhance the understanding of OCCC in the context of pregnancy.

## 2. Case reports

A 33-year-old female patient presented to our hospital for fertility preservation treatment with a 14-week history of amenorrhea and a pelvic mass detected by ultrasound 2 months prior. Ultrasonographic examination reveals an intrauterine early pregnancy with viable embryo. A mixed echogenic mass measuring approximately 8.6 × 6.6 × 6.2 cm is observed in the left adnexal region, with relatively well-defined borders. Within this mass, an irregular hypoechoic area measuring 4.5 × 3.5 cm with moderate echogenicity and minimal blood flow signals is identified. Additionally, thrombosis is detected in the left superficial branch of the peroneal vein and the right intermuscular vein of the calf.

Laboratory tests demonstrate elevated tumor markers: CA-125 (737.11 U/mL) and CA724 (>250.00 U/mL), while other markers remain within normal ranges. Under spinal anesthesia, unilateral salpingo-oophorectomy and pelvic adhesiolysis are performed. Intraoperative findings reveal a diffusely enlarged uterus consistent with gestational size at 12 to 14 weeks, and a left ovarian enlargement (approximately fist-sized) adherent to the pelvic wall. The omentum and portions of the intestine are found adhered to the left pelvic wall, while the right adnexa appears normal.

The patient’s surgical history includes a cesarean section 2 years prior (gravida 2, para 1), with no personal or family history of malignancy. Retrospective review of the patient medical records indicates that during the first pregnancy 2 years ago, a 2.0 cm cystic lesion in the left adnexa was detected on ultrasound and presumed to be an endometriotic cyst. This lesion has demonstrated gradual enlargement over the past 2 years, with accelerated growth during the current pregnancy.

**Figure 1.**
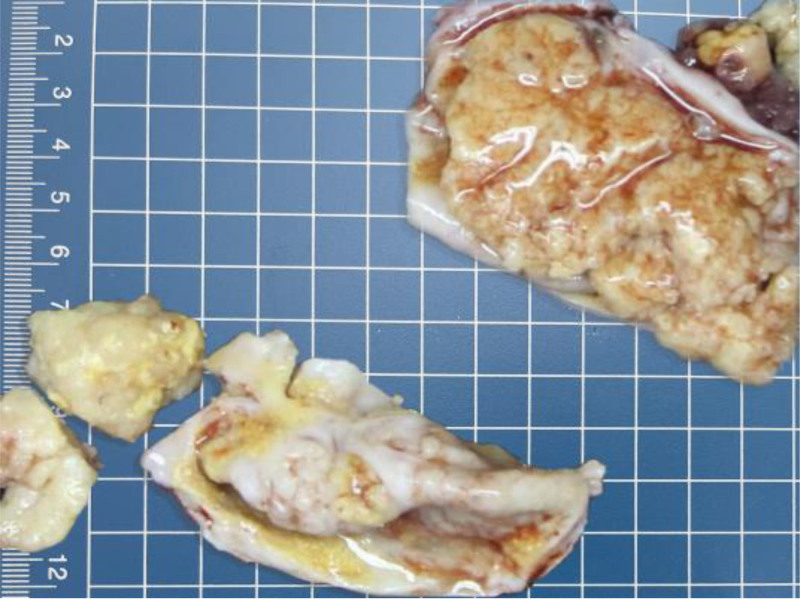
A grayish-yellow cystic-solid mass with a papillary surface is observed.

Gross examination reveals a 4-cm-long fallopian tube (diameter: 0.5 cm) and a cystic-solid mass measuring 7.5 × 4 × 3 cm, exhibiting a grayish-yellow, papillary surface (Fig. 1). The cyst wall and solid components are sampled for intraoperative frozen section analysis.

**Figure 2.**
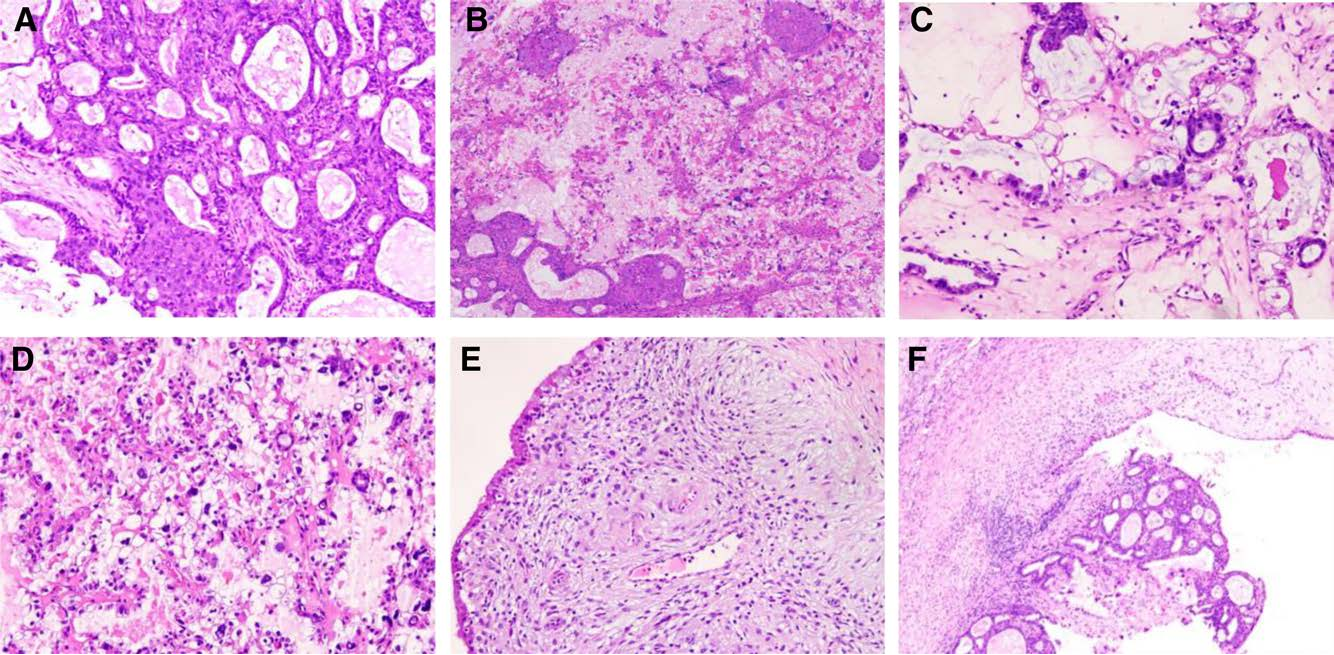
(A–B) The tumor exhibits adenotubular architecture with squamous differentiation (×200). (C) Hyalinized fibrovascular cores are seen within papillary structures (×200). (D) Tumor cells display clear cytoplasm and hobnail morphology (×200). (E) Endometriotic cysts with pregnancy-related alterations were identified adjacent to the tumor (×200). (F) The pathological progression from endometriosis to atypical endometriosis and carcinoma is demonstrated (×100).

**Figure 3.**
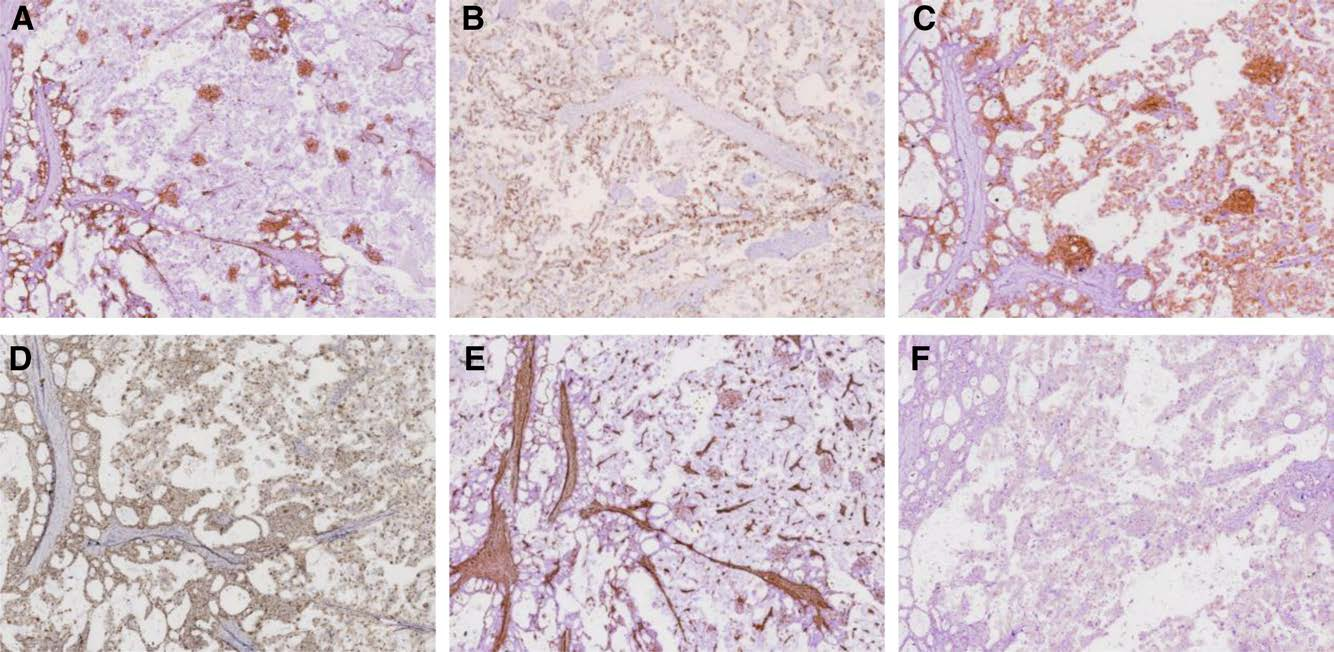
(A) CD10 expression in the squamous differentiation area by the EnVision method (×200). (B) Cytoplasmic NapsinA positivity is detected by the EnVision method (×200). (C) Cytoplasmic P504S positivity is detected by the EnVision method (×200). (D) Nuclear WT1 positivity is detected by the EnVision method (×200). (E) Tumor cell VIM negativity is detected by the EnVision method (×200). (F) Focal weak cytoplasmic positivity for P53 is detected in tumor cells by the EnVision method. (×200).

Microscopically, the tumor demonstrates cystic, papillary, and solid growth patterns, with prominent squamous differentiation within glandular structures (Fig. 2A–B). Papillary regions feature hyalinized fibrovascular cores lined by cuboidal cells with clear cytoplasm (Fig. 2C), hobnail morphology, marked nuclear pleomorphism, and hyperchromasia (Fig. 2D). Endometriotic cysts with pregnancy-related alterations were identified adjacent to the tumor (Fig. 2E), alongside a transition from endometriosis to atypical endometriosis and carcinoma (Fig. 2F). CD10 expression in the squamous differentiation area (Fig. 3A), Tumor cells show positivity for CK7, EMA, NapsinA (Fig. 3B), P504S (Fig. 3C), and WT1 (Fig. 3D). Focal estrogen receptor/progesterone receptor expression, patchy p16 positivity, while SALL-4, α-inhibin, Vimentin (Fig. 3E), and thyroid transcription factor-1 are negative. Focal weak cytoplasmic positivity for p53 is observed in tumor cells (Fig. 3F). The Ki-67 proliferation index is approximately 20%.

Intraoperative frozen section: Suspicious for sex cord-stromal tumor; deferred to permanent pathology. Final diagnosis: OCCC.

## 3. Discussion

The fifth edition of the World Health Organization classification of ovarian tumors includes clear cell carcinoma, endometrioid carcinoma, mucinous carcinoma, as well as high-grade and low-grade serous carcinomas. OCCC is classified as an epithelial ovarian malignancy with a relatively low incidence, accounting for approximately 5% to 10% of epithelial ovarian tumors.^[1]^ Notably, the occurrence of ovarian malignancies during pregnancy is exceptionally rare, with reported incidence rates of 0.075% to 0.110%.^[2]^

The clinical presentation of pregnancy-associated ovarian cancer is usually nonspecific. Thrombophilia or thromboembolic complications may occur and can serve as the initial manifestation prompting medical evaluation. Most cases are diagnosed at advanced stages. Ultrasonography represents the primary imaging modality for diagnosis during pregnancy,^[4]^ particularly as routine prenatal ultrasound examinations provide opportunities for detection. Suspicious sonographic features include tumor diameter > 6 cm, abundant intralesional vascularity, cyst wall thickening, and solid components with cauliflower-like or papillary architectures,^[5]^ while serum CA125 levels are typically elevated in ovarian cancer, interpretation during pregnancy requires caution due to physiological CA125 elevation. Values ≥ 60 kU/L may have clinical significance, though the diagnostic utility of CA125 monitoring in pregnancy remains limited.^[6]^

OCCC demonstrates a well-established association with ovarian endometriosis.^[7]^ Both endometriosis and clear cell borderline tumor are recognized precursor lesions. In the present case, endometriotic cysts were initially suspected during the patient’s first pregnancy, with definitive OCCC diagnosis established during the second pregnancy via pathological examination. Histological evaluation demonstrates the malignant progression from endometriosis through atypical endometriosis to carcinoma.

The pathological diagnosis of OCCC relies on characteristic morphological features, including 4 growth patterns: tubular, papillary, cystic, and solid, and 2 cell types: hobnail cells and clear cells, with the presence of hyalinized stroma.

The main differential diagnoses include endometrioid carcinoma-secretory subtype: OCCC is associated with poorer prognosis compared to endometrioid carcinoma,^[8]^ making accurate diagnosis prognostically significant. Histological overlap occurs when secretory-type endometrioid carcinoma during pregnancy demonstrates cytoplasmic clearing from squamous or secretory differentiation, resembling OCCC, particularly as both may arise from endometriosis. Endometrioid carcinoma is characterized by highly columnar tumor cells with subnuclear/nuclear vacuoles resembling early secretory endometrium, while OCCC exhibits greater nuclear heterogeneity with high-grade nuclei. Immunohistochemical markers hepatocyte nuclear factor 1 beta and NapsinA show specificity for OCCC,^[9]^ with NapsinA being particularly diagnostic. The current case demonstrates marked nuclear atypia with hyalinized fibrovascular cores and supportive immunohistochemistry confirming OCCC. Mesonephric adenocarcinoma displays diverse histological patterns including tubular, papillary, glandular, tubulocystic and solid architectures, featuring eosinophilic intraluminal secretions and cells with round/oval nuclei, coarse chromatin, small nucleoli, and variably eosinophilic or clear cytoplasm. Immunohistochemical expression of thyroid transcription factor-1 and GATA3 aids differentiation. High-grade serous carcinoma with clear cell features requires distinction from OCCC, though the latter typically contains hyalinized stroma versus fibrotic/edematous changes in serous carcinoma. While OCCC commonly shows papillary/cystic patterns, high-grade serous carcinoma typically exhibits WT-1, ER, and PR positivity with mutant p53 expression. Endometrioid and clear cell mixed carcinoma: The majority of this specimen exhibits typical clear cell carcinoma, with focal areas showing glandular patterns accompanied by squamous differentiation. The possibility of endometrioid and clear cell mixed carcinoma should be considered. However, since the specimen did not express the corresponding immunohistochemical markers for the different carcinoma types, the final diagnosis is clear cell carcinoma with squamous differentiation.

Although elevated estrogen levels are known to promote ovarian cancer while progesterone exerts an inhibitory effect,^[3]^ epidemiologic studies demonstrate an inverse association between multiparity and ovarian cancer, with not only reduced incidence but also delayed onset in multiparous women.^[10]^ However, in this case, tumor progression was markedly accelerated following the second pregnancy. Given the rarity of primary ovarian malignancies during pregnancy, no clear or standardized treatment protocol exists. Current clinical management options include standard ovarian cancer treatment following pregnancy termination; conservative surgery with subsequent chemotherapy, followed by definitive staging surgery after delivery; or neoadjuvant chemotherapy during pregnancy, followed by radical surgery postpartum.^[11]^ In this patient, total hysterectomy, bilateral adnexectomy, and pelvic lymph node dissection were performed after pregnancy termination, considering the patient’s age and reproductive desires. The clinical stage and histologic grade of pregnancy-associated ovarian malignancies are strongly correlated with prognosis. Although ovarian cancer during pregnancy is exceedingly rare, pregnancy-related ovarian malignancies exhibit a more favorable prognosis compared to non-pregnancy-associated cases,^[12]^ and survival is not influenced by gestational or postpartum status.^[12]^ Furthermore, existing literature reports no significant reduction in survival rates.^[13]^ In the present case, the patient underwent surgery and was followed for 6 months postoperatively, with no recurrence detected to date. However, the relatively short follow-up period may limit the assessment of recurrence risk and long-term prognosis. Continued follow-up is warranted.

## 4. Conclusion

In conclusion, while an inverse correlation is established between progesterone exposure, pregnancy history, and ovarian cancer development, this rare case demonstrates OCCC occurring during a second pregnancy, exhibiting squamous differentiation mimicking endometrioid carcinoma histologically. OCCC, characterized by poorer prognosis compared to endometrioid carcinoma, is pathomorphologically distinguished in this case by its documented progression from endometriosis through atypical endometriosis to malignancy. This neoplastic transformation is frequently associated with thrombotic disorders or embolic complications, which may serve as the initial clinical manifestation. Consequently, neither benign-appearing cysts nor thrombotic events during pregnancy should be disregarded, as they may represent early indicators of underlying malignancy requiring thorough evaluation.

## Author contributions

**Project administration:** Quan Chen, YinXia Su.

**Writing – original draft:** Yong Wang.

**Writing – review & editing:** Yong Wang, Fei Liu.
